# Mast Cell/Proteinase Activated Receptor 2 (PAR2) Mediated Interactions in the Pathogenesis of Discogenic Back Pain

**DOI:** 10.3389/fncel.2019.00294

**Published:** 2019-07-05

**Authors:** Justin Richards, Shirley Tang, Gilian Gunsch, Pavel Sul, Matthew Wiet, David C. Flanigan, Safdar N. Khan, Sarah Moore, Benjamin Walter, Devina Purmessur

**Affiliations:** ^1^College of Arts and Sciences, The Ohio State University, Columbus, OH, United States; ^2^College of Engineering, The Ohio State University, Columbus, OH, United States; ^3^Department of Orthopaedics, Wexner Medical Center, The Ohio State University, Columbus, OH, United States; ^4^College of Veterinary Medicine, The Ohio State University, Columbus, OH, United States

**Keywords:** mast cell, tryptase, PAR2, intervertebral disc, discogenic back pain

## Abstract

Mast cells (MCs) are present in the painful degenerate human intervertebral disc (IVD) and are associated with disease pathogenesis. MCs release granules containing enzymatic and inflammatory factors in response to stimulants or allergens. The serine protease, tryptase, is unique to MCs and its activation of the G-protein coupled receptor, Protease Activated Receptor 2 (PAR2), induces inflammation and degradation in osteoarthritic cartilage. Our previously published work has demonstrated increased levels of MC marker tryptase in IVD samples from discogenic back pain patients compared to healthy control IVD samples including expression of chemotactic agents that may facilitate MC migration into the IVD. To further elucidate MCs’ role in the IVD and mechanisms underlying its effects, we investigated whether (1) human IVD cells can promote MC migration, (2) MC tryptase can mediate up-regulation of inflammatory/catabolic process in human IVD cells and tissue, and (3) the potential of PAR2 antagonist to function as a therapeutic drug in *in vitro* human and *ex vivo* bovine pilot models of disease. MC migration was quantitatively assessed using conditioned media from primary human IVD cells and MC migration examined through Matrigel. Exposure to soluble IVD factors significantly enhanced MC migration, suggesting IVD cells can recruit MCs. We also demonstrated significant upregulation of MC chemokine SCF and angiogenic factor VEGFA gene expression in human IVD cells *in vitro* in response to recombinant human tryptase, suggesting tryptase can enhance recruitment of MCs and promotion of angiogenesis into the usually avascular IVD. Furthermore, tryptase can degrade proteoglycans in IVD tissue as demonstrated by significant increases in glycosaminoglycans released into surrounding media. This can create a catabolic microenvironment compromising structural integrity and facilitating vascular migration usually inhibited by the anti-angiogenic IVD matrix. Finally, as a “proof of concept” study, we examined the therapeutic potential of PAR2 antagonist (PAR2A) on human IVD cells and bovine organ culture IVD model. While preliminary data shows promise and points toward structural restoration of the bovine IVD including down-regulation of VEGFA, effects of PAR2 antagonist on human IVD cells differ between gender and donors suggesting that further validation is required with larger cohorts of human specimens.

## Introduction

Approximately 70–80% of the population will experience chronic low back pain during their lifetime (Andersson, 1999) and large population-based studies have demonstrated that intervertebral disc (IVD) degeneration is a significant cause of chronic low back pain (De Schepper et al., 2010). The huge socioeconomic burden of Discogenic Back Pain (DBP) is not only a result of widespread use of interventions that are costly with limited efficacy, but also the role of DBP in the growing opioid epidemic, as opioids are the most widely prescribed drug used to treat back pain (Balagué et al., 2012; Deyo et al., 2015). Initial DBP therapies are largely conservative and include analgesics, physiotherapy and psychosocial pain management approaches. When these approaches are unsuccessful, highly invasive surgeries (e.g., disc arthroplasty or lumbar fusions) are routinely performed. Yet, these treatments are often short-lived, fail to target the underlying cause of disease and can significantly reduce patient mobility often leading to adjacent segment disc disease (Mannion et al., 2014).

The healthy IVD is characterized by a healthy gelatinous core of proteoglycans and nucleus pulposus (NP) cells of notochordal origin. This NP core is enclosed radially by concentric rings of collagen to form the annulus fibrosus (AF) with fibroblast-like AF cells aligned with the collagen fibrils. The joint is encased caudally and cranially by the cartilage end plate (CEP), hyaline cartilage crucial to bidirectional diffusion of nutrients and metabolites and separates the IVD from the vertebral bodies (Roughley, 2004). Degeneration of the IVD is characterized by significant increases in extracellular matrix (ECM) breakdown, inflammation and nerve/vascular ingrowth (Smith et al., 2011; Lama et al., 2018). Nociceptive neurons and blood vessels expressing nerve growth factor (NGF) have been demonstrated in the painful human IVD (Freemont et al., 1997, 2002), specifically in regions of ECM depletion and injury (Stefanakis et al., 2012; Lama et al., 2018). Our previous work has demonstrated that disc ECM and injury can regulate nerve/vascular growth *in vitro* and *in vivo* (Johnson et al., 2002, 2005; Kim et al., 2011; Purmessur et al., 2015). Additionally, pro-inflammatory cytokines interleukin 1-Beta (IL-1β) and tumor necrosis factor alpha (TNFα), (increased in the degenerate IVD) can up-regulate catabolic enzymes along with neurotrophic and angiogenic factors NGF, vascular endothelial growth factor A (VEGFA), interleukin-6 (IL-6) and substance P in IVD cells (Le Maitre et al., 2005; Séguin et al., 2005; Purmessur et al., 2013a). While the healthy IVD is immune-privileged, increased numbers of innate immune cells have been demonstrated within painful human IVDs compared to healthy IVDs, yet their role within the painful degenerate disc remains unknown (Wiet et al., 2017).

Immune cells play an integral role in tissue remodeling and healing. However, due to the avascular nature of the IVD, repair is commonly described as “frustrated healing” and lacks the native ECM components to restore structure and function (Adams and Roughley, 2006). Innate immune cells, such as mast cells (MCs) and macrophages, have been identified in degenerative human IVD tissue and induce a catabolic and inflammatory phenotype in IVD cells, however, the specific mechanisms underlying these effects remains to be elucidated (Wiet et al., 2017; Nakazawa et al., 2018). MCs function to release preformed granules with enzymatic (tryptase and a disintegrin and metalloproteinase with a thrombospondin motif 5, ADAMTS5) and inflammatory/pain-associated factors (IL-1β, TNFα, VEGFA, NGF and Substance P among others) in response to microenvironmental stimuli or allergens (De Schepper et al., 2010). Furthermore, MCs are active and significantly up-regulated in chronic pain conditions such as migraines, irritable bowel syndrome, rheumatoid arthritis and osteoarthritis (OA) (O’Sullivan et al., 2000; Nigrovic and Lee, 2005; Theoharides et al., 2005; Nakano et al., 2007). In OA, MC-derived mediators enhance mechanical hypersensitivity in nociceptors within the joint space (Sousa-Valente et al., 2018). Macrophages have also been identified in the IVD, however, they do not contain preformed granules but synthesize inflammatory cytokines upon activation [TNFα, IL-1β and prostaglandins (PGE2)] (Gordon and Taylor, 2005; Nakazawa et al., 2018). Similar to MCs, they are present in arthritic diseases and associated with matrix remodeling in disc herniation (Koike et al., 2003; Kobayashi et al., 2009). However, recent studies have demonstrated that macrophages do not directly regulate pain-associated growth factors VEGFA and NGF (Miyagi et al., 2018).

The serine proteinase tryptase is produced by MCs, and its activation of the G-protein coupled receptor, Proteinase Activated Receptor 2 (PAR2; F2RL1), has been shown to induce inflammation and catabolism in human osteoarthritic cartilage (Balagué et al., 2012). PAR2 initiates and enhances painful responses in inflammatory, visceral, and cancer-related pathologies and recent studies demonstrate its activation is sufficient to induce transition to a chronic pain state (Tillu et al., 2015). PAR2 is expressed by multiple cell types including immune cells, nerves and endothelial blood vessel cells, chondrocytes and IVD cells (Klarenbach et al., 2003; Iida et al., 2009; Chen et al., 2011). However, its specific role in DBP remains unknown. PAR2 is also involved in sensitization of neuropeptide receptors (Neurokinin 1) and ion channels (i.e., transient receptor potential cation channel subfamily V member 1; TRPV1) expressed on dorsal root ganglion (DRG) cells, which enhance hyperalgesia and pain (Vergnolle et al., 2001; Amadesi, 2004). Studies by Iida et al. (2009) have demonstrated that PAR2 is expressed in the rat IVD, elevated in painful human IVDs and that its activation upregulates the expression of IL-1β and ADAMTS4 in rat IVD cells. However, whether MC mediators (i.e., tryptase) can activate such inflammatory pathways in human IVD cells is currently unknown and is the focus of the studies described here.

**FIGURE 1:**
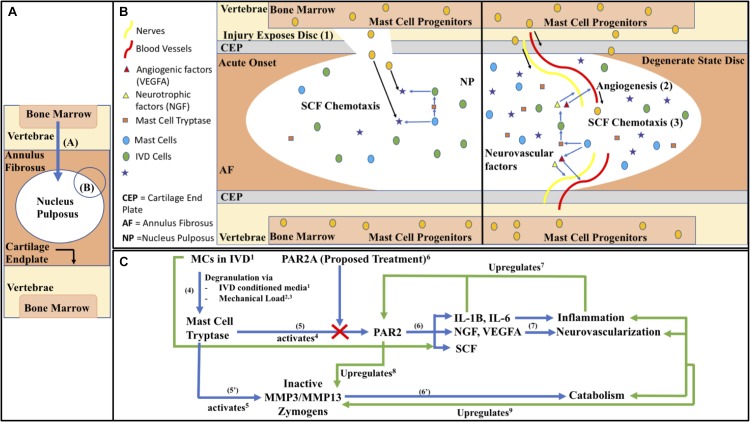
**(A)** Macroscopic IVD schematic with zoomed in proposed mechanistic models of **(B)** MC invasion into the intradiscal environment in acute onset and chronic state as visualized through (1) Acute injury or matrix perturbation provides physical access of MC progenitors into usually immune-privileged intra-discal microenvironment acutely and (2) in the chronic state access can occur through vascularization, (3) facilitated by the MC chemoattractant SCF (KITLG). **(C)** Biochemical pathways of immune modulation in DBP as follows; (4) MCs in the IVD respond to mechanical load and nutrient deprivation in the harsh microenvironment by releasing tryptase (Wiet et al., 2017^1^; Zhang et al., 2012^2^; Fowlkes et al., 2013^3^). Tryptase can activate (5) PAR2 directly (Molino et al., 1997^4^) as well as (5’) matrix enzymes, matrix metalloproteinases 3 and 13 (MMPs) (Magarinos et al., 2013^5^). (6) PAR2 activation by tryptase, abated by PAR2A (Chen et al., 2011^6^) in the present study, leads to downstream factors related to catabolism, (6’) such as MMPs (Lee et al., 2010^8^), which can degrade the proteoglycan aggrecan (Magarinos et al., 2013^5^) creating an increasingly permissive environment for inflammation and stimulating increased activation of endogenous pro-MMP (Vo et al., 2013^9^). (7) PAR2’s activation also leads to downstream factors related to inflammation, neo-vascularization/neo-innervation, and MC chemotaxis contributing to a synergistic positive feedback loop, whereas PAR2’s activation upregulates its own expression through IL-1β, ad general inflammation, perpetuating a chronic degenerate state (Ritchie et al., 2007^7^). Black Arrows represent labeling or directionality, Blue arrows represent forward mechanism steps, Green arrows represent feedback interactions. **(C)** Blunt arrow ends represent effects coming from, pointed ends represent effects going to, and bidirectional pointed ends represent co-regulatory interactions.

The objectives of this study are based on the following hypothetical model as described in Figure 1. Briefly, MC progenitors invade the IVD via injury or matrix perturbation, being specifically recruited to the IVD region from bone marrow, following invasion the IVD microenvironment causes degranulation by the matured MCs and the action of the released mediators either directly on tissue or indirectly via induction of genetic changes on cells, promoting neurovascularization, inflammation, catabolism and ultimately pain. This process is self-perpetuating as elaborated on in Figure 1; MC migration is enhanced directly and indirectly through IVD cells, allowing this degenerative process to repeat and progress into a chronic degenerative state, with the eventual compromised state becoming further conducive to the aforementioned markers of a painful IVD. We hypothesize that, due to these intrinsically synergistic mechanisms, inhibition with the PAR2 antagonist oligopeptide FSLLRY-NH_2_ (PAR2A) (Chen et al., 2011) possesses therapeutic potential to prevent progression of a chronic inflammatory, catabolic and painful state.

This study aims to determine the potential mechanism by which MCs may invade the IVD joint, investigate tryptase effects on matrix degradation and chemotactic/vascular markers, quantify and validate protein expression of PAR2 in NP, AF and CEP regions of the human IVD via immunohistochemistry and western blot and to determine the therapeutic potential of PAR2A in a bovine *ex vivo* translational model and in human *in vitro* model for proof of concept.

## Materials and Methods

### Tissue Procurement and Histological Processing

Mild to moderately degenerate human cadaveric IVD samples (Table 1) were acquired via the Co-operative human tissue network, Midwestern Division (Columbus, Ohio) with institutional IRB exemption. Cadaveric specimens were dissected within 36 h post-mortem and tissue either isolated for cells or histology. For histology, 2 mm (superior to inferior) sagittal sections were collected using a diamond coated band saw. These sections were then fixed in 10% neutral buffered formalin before paraffin embedding for IHC. Human articular cartilage samples (Table 1) were acquired from patients with informed consent in accordance with The Ohio State University (Columbus, Ohio) IRB relevant guidelines and regulations under IRB# 2018H0424.

**Table 1:** Human tissue and cells.

Level/Joint (tissue + cells)	Region (tissue + cells)	Age	Gender
L3-L4; L4-L5	NP, AF, CEP	43	F
L1-L2; L5-S1	NP, AF, CEP	55	M
L3-L4; L4-L5	NP, CEP	45	M
L3-L4; L4-L5	NP, AF, CEP	56	F
L3-L4; L4-L5	NP, AF, CEP	52	M
L2-L3; L3-L4	NP, AF	58	F
L2-L3; L3-L4	NP, AF	46	M
L3-L4	NP, AF	31	F
L4-L5	NP, AF, CEP	30	F
L3-L4	NP, AF	37	F
L4-L5	NP, AF, CEP	59	F
L3-L4	NP	19	F
L3-L4	NP	39	M
Knee	Articular Cartilage	22	F
Knee	Articular Cartilage	20	F
Knee	Articular Cartilage	22	F

### Primary IVD and Articular Cartilage Cells

All primary human IVD and articular cartilage cells (Table 1) were obtained from cadaveric samples, and patients undergoing treatment for osteoarthritis of the knee, respectively, sourced as described in Tissue Procurement. Exclusion criteria was applied for IVD samples, specifically, no infectious disease, discs were intact, no discs from spines older than 60 years old and Thompson scale grade not exceeding 3.5 (discs were blinded, graded by authors independently, and grades averaged). Articular cartilage samples were obtained from regions of intact healthy cartilage with microscopic and clinical assessment by Dr. David Flanigan. Cells were isolated using protease from *Streptomyces griseus* (0.05 mg/25 mL) in digestion media (high glucose (4.5 g/mL) DMEM, 1% Penicillin/Streptomycin (P/S), 0.5% Fungizone) for 1 h before 12 h digestion in Collagenase I or II (0.003 g/25 mL) for AF or NP tissue, respectively as cited previously (Purmessur et al., 2011). Following isolation, cells were expanded in Disc Cell Complete media (DCC; high glucose DMEM, 10% FBS, 1% P/S, 50 μg/mL Ascorbic Acid) in standard conditions (37°C, 20% O_2_, 5% CO_2_) and fed once every 3 days until ∼90% confluent. All cells used for *in vitro* studies were at passage < P4.

### Mast Cell Culture

Human Mast Cell line 1 (HMC-1) cells were a generous gift of Dr. J.H. Butterfield (Mayo Clinic). HMC-1 cells were expanded in suspension in Basal HMC-1 media (BMC; Iscove’s modified dulbecco’s medium, 10% FBS, 1% P/S, 1.2 mM α-thioglycerol) in standard conditions (37°C 20%O_2_ 5%CO_2_) and fed 2–3 times a week until experimental use. The HMC-1 cell line is a well characterized and thoroughly validated human MC line (Nilsson et al., 1994a), however, as this is an immortalized cell line, they contain a c-kit mutation that allows for easier handling and survivability. It has been shown that these cells may have reduced tryptase levels when compared to mature human skin MCs (Guhl et al., 2010).

### Generation of 100% MCCM

HMC-1 cells were expanded as described above. To create mast cell conditioned media (MCCM) cells were diluted to 5.0 × 10^5^ cells/mL in BMC and frozen for 24 h at –80°C. Following freezing, the cell suspension was thawed quickly in a 37°C water bath to maximize cell lysis. The resulting cell lysate solution was centrifuged at 1000 × *g* for 5 min to pellet cells and the supernatant collected and filtered using Pall Centrifugal devices (Pall Corp. MAP003C37) at 3000 × *g* for 30 min followed by reconstitution to the original volume with BMC. This final solution represents 100% MCCM including MC related factors such as MC tryptase. This 100% MCCM was then diluted to the desired 50% MCCM with DCC. Viability was assessed via trypan blue uptake before and after lysis. Protein concentration was assessed using a Bradford assay (Bio-Rad protein assay kit) for conditioned media at pre-lysis, post-lysis and post-filtration steps (Bradford, 1976).

### Mast Cell Invasion/Migration in Response to IVD Related Factors

HMC-1 cells were expanded as described in MC culture. The cell invasion/migration assay was performed as cited previously (Brekhman and Neufeld, 2012). Briefly, Matrigel was diluted to 800 μg/mL in Iscove’s modified Dulbecco’s medium and used to coat the migration insert. HMC-1 cells were diluted to 9.0 × 10^5^ in 0% FBS BMC immediately prior to use. Intervertebral disc conditioned media (IVDCM) (*N* = 3) and articular cartilage conditioned media (ACCM) (*N* = 3) was generated by culturing primary cells from cadaveric human IVD NP or articular cartilage cell samples at 1.0 × 10^6^ cells/mL in 0% FBS DCC for 48 h before collecting this media for use. HMC-1 samples were tested in triplicate for migration with experimental conditions of 0% FBS BMC (negative control), 0% FBS IVDCM, 0% FBS ACCM (Cell Control), or 10% FBS BMC (positive control) added to the bottom of the well. Migrated live MCs were quantified via staining the bottom surface of the membrane with fluorescent dye Calcein (4 μM for 15 min at 37°C), staining all live cells green, and imaged by Nikon-Eclipse inverted microscope. Images were captured at 4× magnification and blinded prior to quantification. The number of migrated MCs were quantified automated via Nikon Analysis Software for each group and normalized to the 10% FBS BMC group (positive control) as a percent migration, whereas 10% FBS BMC represents 100% migration.

### MCCM/IVD Interactions and the PAR2 Pathway

#### Tryptase Effects on IVD

##### Cells

Primary human AF and NP cells from cadaveric human tissue (*N* = 4; Table 1) were seeded in 2% agarose gels using a silicone mold at 4.0 × 10^6^ cells/mL (1.6 × 10^6^ cells/construct) for 24 h in DCC+10%FBS media at 37°C 20%O_2_ 5%CO_2_, followed by 24 h in DCC+2.5% FBS at 37°C 5%CO_2_ 5%O_2_ with 0.00, 0.01, or 0.10 μg/mL (Masuko et al., 2007) of purified recombinant human tryptase from lung MCs (rhTryptase; Promega G5631). One quadrant of each construct was used to assessed viability (4 μM Calcein + 2 μM ethidium for 15 min at 37°C). The remaining portion of each construct was homogenized in TRIzol and mixed with 0.2 mL chloroform/mL TRIzol. This mixture was centrifuged at 12,000 × *g* for 15 min at 4°C to collect the aqueous upper phase and diluted 1:1 with molecular grade 70% EtOH before mRNA purification using the PuraLink RNA mini kit following manufacturer instructions (Life Technologies) for downstream cDNA synthesis with Maximus H Minus (Thermo Fisher Scientific M1662) and gene expression (qRT-PCR) for MC related chemoattractant SCF and angiogenic marker VEGFA (Table 2). Gene expression was quantified using the comparative ΔΔCt method (Livak and Schmittgen, 2001) with levels normalized to the housekeeping gene 18s (Table 2).

**Table 2:** Gene assays.

Gene	Assay ID
PAR2 (F2RL1)	Hs00608346_m1
SCF (KITLG)	Hs00241497_m1
VEGFA	Hs00900055_m1; Bt03213282
NGF	Hs00171458_m1
IL-1B	Hs01555410_m1
IL-6	Hs00174131_m1
MMP3	Hs00968305_m1
MMP13	Hs00942584_m1

##### Tissue

Human cadaveric IVD samples (*N* = 8; Table 1) were freeze-thawed and 8 mm biopsy samples of NP and AF tissue were collected, wet-weight measured, and pre-incubated for 24 h in basal DMEM at 37°C, 20%O_2_, 5%CO_2_ to account for endogenous aggrecan loss. Non-live tissue (by means of freeze thaw) was utilized to examine the isolated effects of tryptase on proteoglycan accumulation in the absence of any cellular effects. Samples were then incubated with fresh basal media supplemented with 0.0, 1.0, or 5.0 μg/mL rhTryptase (adapted from Magarinos et al., 2013) for 48 h at 37°C 20%O_2_ 5%CO_2_. Information on genes pertaining to our results can be found in Supplementary Table S1. Endogenous proteoglycan degradation was measured by sulfated glycosaminoglycan (GAG) release into media indicated by the colorimetric dimethylmethylene blue assay (DMMB; Sigma-Aldrich 341088) with absorbance read at 530 nm. A standard curve using shark chondroitin sulfate from shark cartilage (Sigma-Aldrich C4384) in DMEM with a linear regression analysis *R*^2^ value > 0.95 was used to calculate concentration from absorbance value.

### PAR2 Expression in Human IVD Tissue

#### Immunohistochemistry

Human cadaveric IVD samples (Table 1) of AF (*N* = 6), NP (*N* = 9) and CEP (*N* = 7) were assessed via IHC using SAM11, a primary antibody for PAR2 (1:50; Invitrogen 35-2300). Briefly, tissue slides were deparaffinized, rehydrated, blocked for endogenous peroxidase activity (0.3% H_2_O_2_ in MeOH), and antigens retrieved using a citrate buffer (90°C, pH 6.0) for 20 min. Blocking for non-specific binding was performed with 5% goat-serum (1% BSA-PBS, 5% goat serum, 0.05% Tween, and 0.05% sodium azide). Primary antibodies were incubated for 2.5 h in background reducing antibody diluent (Dako S3022) followed by incubation with secondary antibody: biotinylated goat anti-mouse (1:200, VectorLabs BA9200). Finally, tissue sections were incubated with streptavidin-horse radish peroxidase and developed with 3,3-diaminobenzidine (DAB) for 90 s (VectorLabs SK-4100). Tissue was counterstained with Gills No. 2 Hematoxylin (Electron Microscopy Sciences 26030-20) and slides dehydrated and mounted. Normal human lung tissue was used for positive and negative controls, with omission of primary antibody for the latter. All antibody concentrations and DAB times were kept consistent across experimental and control samples. Each tissue sample was quantified as described prior (Wiet et al., 2017).

#### Western Blot

To confirm protein expression of PAR2 in IVD cells western blot was performed as described previously (Walter et al., 2016) for cadaveric IVD cell lysate samples from NP, AF, and CEP cells (*N* = 2). PAR2 specific antibody SAM11 (1:250; Invitrogen 35-2300) was used with secondary antibody biotinylated goat-anti mouse (1:1000; VectorLabs BA-9200) and run with positive control for β-actin (ACTB; 1:5000; abcam ab8227).

### PAR2A Therapeutic Proof of Concept Pilot *in vitro* Human and *ex vivo* Bovine Studies

#### Bovine *ex vivo* Therapeutic Model

A proof of concept study was performed to evaluate the potential effects of PAR2A on matrix degradation and angiogenesis. Skeletally mature bovine coccygeal motion segments (*N* = 3) were cleaned of soft tissue and IVD isolation was performed using a diamond coated band saw leaving 1–3 mm of CEP/Vertebral body caudally and cranially for 3 IVDs per bovine sample. Isolated samples were washed in 70% EtOH and PBS before being treated with isolation (Prime Growth 319-511-EL) and neutralizing solution (Prime Growth 319-512-CL) and then culture in Wisent Prime Growth Media (Prime Growth 319-510-CL) + 1% P/S for 24 h at 37°C 5% CO_2_ 20% O_2_ according to Wisent protocol as outlined prior (Grant et al., 2016). Injury was induced for relevant samples via an “X” cut through the AF region and partial NP removal (Illien-Jünger et al., 2014). After 10 days, samples were split into the following groups: (1) control with no injury or treatment, (2) injury+MCCM, or (3) injury+MCCM+100 nM PAR2A. 10 μL of 100 nM PAR2A (FSLLRY-NH_2_) in PBS was injected with a high-precision 25G syringe (Hamilton; Reno, NV, United States) into the relevant bovine IVDs (Illien-Jünger et al., 2014). IVDs were cultured for 96 h and then tissue isolated for histology, cell viability and qRT-PCR. Briefly, the IVD was cut into 3 × 2 mm in width sagittal sections with a ceramic band. Of the three sections the medial segment, which included the site of injury, was assessed for histology with the lateral portions used for viability and gene expression. For gene expression, AF and NP regions were isolated before being flash-frozen in liquid nitrogen and homogenized via crushing with metal rods, washed in 70% EtOH, RNase Zap (Thermo Fisher Scientific AM9780) and cooled in liquid nitrogen. Tissue samples were dissolved, RNA purified, and qRT-PCR performed as described previously for VEGFA to assess the ability of PAR2A for reduction of MC related angiogenic effects (Table 2). To assess explant viability, tissue was incubated in MTT (3-(4,5-Dimethyl-2-thiazolyl)-2,5-diphenyltetrazolium Bromide; Tokyo Chemical Industry D0801) as described prior (Walter et al., 2016). Each specimen was treated in MTT solution (20 mg/10 mL) for 4 h at 37°C 5% CO_2_ 20% O_2_. The specimens were then fixed in 10% neutral buffered formalin for 48 h before being frozen and cryosectioned (10 μm). Slides were then counterstained with Hoechst 33258 (Sigma-Aldrich 94403) solution (1 μg/mL) for 10 min before being mounted and imaged at 40× magnification. For histology tissue was fixed in 10% neutral buffered formalin before being decalcified in EDTA solution for 7 days, then paraffin embedded, sectioned (10 μm), and stained using alcian blue and picrosirius red solutions (Electron Microscopy Sciences 10350; 26357-02). Stitched images of 16 frames at 4× magnification were taken.

#### Human *in vitro* Monolayer Therapeutic Model

To assess the clinical relevance of PAR2A on human cells *in vitro*, human NP cells from cadaveric tissue (*N* = 6; Table 1) were seeded in monolayer in duplicate at 1.5 × 10^5^ cells/mL and incubated in DCC for 24 h at 37°C 5% CO_2_. After 24 h, culture media was removed and replaced with Basal control media, 50% MCCM or 50% MCCM+100 nM PAR2A (Chen et al., 2015) and incubated for 48 h at 37°C 5%CO_2_. One well was assessed for viability (4 μM Calcein + 2 μM ethidium for 10 min at 37°C) and one well was used to assess gene expression and lysed using 300 μL 1% 2-mercaptoethanol in lysis buffer and diluted 1:1 in 70% molecular grade EtOH before RNA was purified using PuraLink RNA mini kit as per manufacturer instructions. Downstream cDNA synthesis and gene expression quantification (qRT-PCR) for inflammatory, catabolic, and migratory factors SCF, VEGFA, NGF, IL-1β, and IL-6 (Table 2) was completed as described previously.

### Statistical Analysis

All tests run with an α = 0.05.

#### MC Migration (*N* = 3 Biological *N* = 6 Experimental)

Non-parametric, unpaired, one-tailed *T*-tests were performed between groups comparing experimental and biological replicates with all data normalized to the 10% FBS positive control group expressed as 100%.

#### Human *in vitro* 3D Model Gene Expression (*N* = 4)

Non-parametric, paired, two-tailed *T*-tests per gene were performed for paired biological replicates comparing fold changes in ΔΔCt gene expression between treatment groups.

#### Matrix Degradation (*N* = 8)

Non-parametric, paired, one-tailed, *T*-tests were performed for biological replicates comparing the GAG released into the media as a measure of proteoglycan degradation.

#### PAR2 Protein Expression (*N* = 6–9)

Non-parametric, unpaired, one-way ANOVA for comparing unpaired biological replicates for the percentage of positively stained cells as a percentage of the total cells.

#### Bovine *ex vivo* Gene Expression (*N* = 3)

Non-parametric, unpaired, two-tailed *T*-tests were performed for biological replicates comparing fold changes in ΔΔCt gene expression of *ex vivo* angiogenic growth factor.

#### Preliminary Human PAR2A Therapeutic Monolayer *in vitro* Model and Demographic Differences (*N* = 6)

Non-parametric, unpaired, two-tailed *T*-tests were performed for biological replicates comparing fold changes in ΔΔCT gene expression of inflammatory and pain-associated markers.

## Results

### MC Migration in Response to IVD Related Factors

**FIGURE 2:**
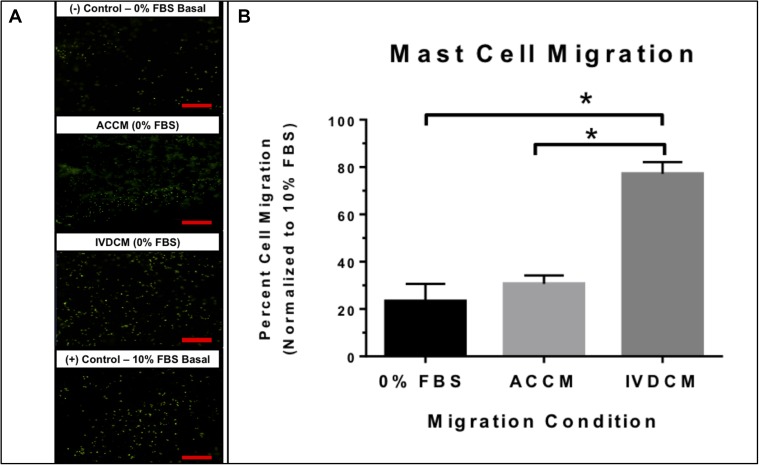
**(A)** Representative images of (calcein) stained fluorescent MCs migrating in response to 0% FBS BMC (negative control), 0% FBS ACCM (cell control), 0% FBS IVDCM, and 10% FBS BMC (positive control). **(B)** Mast cell migration in response to IVDCM (*N* = 3), ACCM (*N* = 3) and 0% FBS (negative control) (*N* = 6) normalized to 10% (positive control = 100% migrated cells) (*N* = 6; ^∗^*p* ≤ 0.05). Red scale bar = 500 μm (4×).

To assess the ability of IVD cell related factors to recruit MCs, a migration assay exposing MCs to IVDCM was performed with ACCM, 0% FBS, and 10% FBS controls. Conditioned media from human NP cells induced migration of MCs through the Matrigel 3D matrix and this migration was significantly greater than that of 0% FBS BMC (negative control) medium, or 0% FBS ACCM (cell control) medium alone (*p* ≤ 0.05) with no significant difference between 0% FBS ACCM and 0% FBS BMC (Figure 2). Data is presented normalized to 10% FBS BMC (positive control) as percent migration with 10% FBS BMC equaling 100%, with an average percentage cell count of 23.3 ± 7.3% for 0% FBS BMC, 30.6 ± 3.6% for 0% FBS ACCM, and 77.1 ± 5.0% for 0% FBS IVDCM as visualized by calcein fluorescent staining of the migrated cells. This data suggests that MCs can migrate in response to IVDCM and more specifically in response to IVD related factors, as the healthy ACCM cell control had little effect compared to negative control.

### Tryptase Effects on IVD Cells (NP and AF)

**FIGURE 3:**
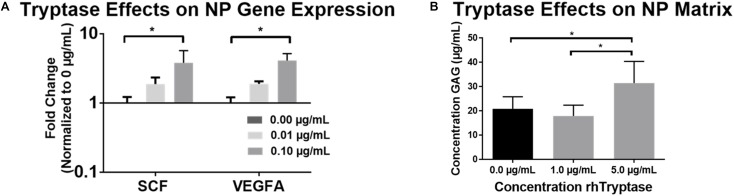
**(A)** Average fold changes in qRT-PCR gene expression in human NP cells (*N* = 4) in 3D agarose constructs treated with rhTryptase and normalized to untreated controls (^∗^*p* < 0.05). **(B)** GAG release into media from freeze-thawed human NP explants from cadaveric (autopsy) IVD specimens (*N* = 8; ^∗^*p* < 0.05).

In order to evaluate the metabolic effects of MC Tryptase on IVD cells *in vitro*, NP and AF cells were treated with rhTryptase in 3D culture and relevant angiogenic (VEGFA) and chemotactic (SCF) factors investigated. rhTryptase at 0.1 μg/mL showed significant upregulation of SCF (3.80-Fold) and VEGFA (4.09-Fold) gene expression relative to untreated control (*p* < 0.05) (Figure 3A). No significant differences in SCF or VEGFA were detected for AF cells (*p* > 0.05) (data not shown). No significant differences in viability were detected in any group (NP or AF). This data is indicative of increased angiogenic/chemotactic potential in IVD cells in response to tryptase.

### Tryptase Effects on IVD Tissue (NP and AF)

To assess the enzymatically degradative effects (direct and or indirect) of tryptase on IVD tissue, tissue explants were treated with rhTryptase. The addition of 5.0 μg/mL rhTryptase significantly increased the GAG released into media from human NP tissue compared to 1.0 and 0 μg/mL rhTryptase (*p* < 0.05) (Figure 3B). No significant differences in GAG release into media were observed in any group from human AF tissue (Supplementary Figure S1). The results suggest that tryptase plays a role in enzymatical degradation of the proteoglycan which may affect structure/function of the IVD tissue.

### PAR2 Expression in Human IVD Tissue

**FIGURE 4:**
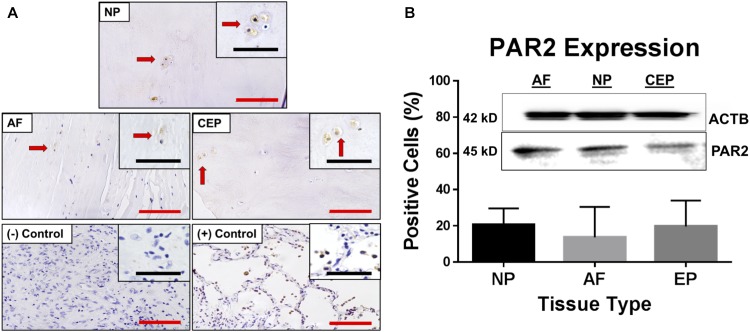
**(A)** Representative images of immunohistochemical (IHC) PAR2 staining in IVD tissue from all regions; NP, AF and CEP including relevant positive and negative lung tissue controls. **(B)** PAR2 expression presented as % of cells expressing PAR2 as assessed by IHC and confirmed with western blot using the same PAR2 primary antibodies. Red scale bar = 100 μm, Black scale bar = 50 μm. Arrows point to same cells at both magnifications.

To evaluate a potential role for PAR2 in mediating the down-stream effects of MCs and tryptase, we first performed IHC for PAR2 on cadaveric human IVD tissue. Protein expression was assessed via IHC for PAR2 in human tissue (% of positive cells) in NP (20.5% ± 9.16), AF (13.6% ± 16.48) and CEP (19.6% ± 14.32) cells with no significant differences detected between regions (Figure 4). PAR2 protein expression was confirmed in the NP, AF and CEP regions of the human IVD via Western blot (Figure 4B and Supplementary Figure S2). This expression validates previously reported PAR2 expression in human IVDs (Iida et al., 2009) and points toward a potential pathway mediating MC effects in the human IVD.

### Bovine *ex vivo* Therapeutic Model

**FIGURE 5:**
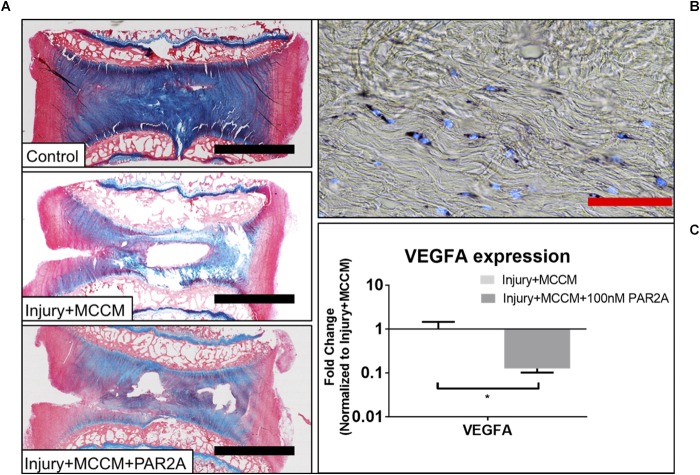
**(A)** Representative Alcian blue (aggrecan) and picrosirius red (collagen) staining of bovine caudal IVDs after 96 h of culture. Discs were either left intact (control) or injured and cultured with MCCM with or with a PAR2A. **(B)** Representative cell viability image for Injury groups treated with MCCM+PAR2A. **(C)** Average fold change in qRT-PCR gene expression in AF region of bovine *ex vivo* samples (^∗^*p* < 0.05). Red scale bar = 1000 μm, Black scale bar = 50 μm.

As a preliminary proof of concept study to assess the potential therapeutic effects of a PAR2 antagonist on MC-induced IVD degeneration, we chose to use a bovine *ex vivo* organ model which has been utilized previously as a model of IVD degeneration and to screen therapeutics (Purmessur et al., 2013b; Illien-Jünger et al., 2014; Grant et al., 2016). Treatment of IVD organ culture samples with PAR2A had no detectable impact on viability for AF as demonstrated by MTT staining co-localized with DNA stain Hoechst (Figure 5B). Due to technical limitations, the NP could not be assessed; However, RNA levels and histology suggest adequate viability due to matrix generation. Treatment with 100 nM PAR2A significantly down-regulated the gene expression of VEGFA (7.87-Fold) in the AF region of MCCM treated injured disc samples relative to MCCM injury controls (*p* < 0.05) (Figure 5C). No significant changes in VEGFA expression were observed in the NP (data not shown). Treatment with PAR2A was also correlated with visibly enhanced tissue regeneration as shown by increased histological staining of proteoglycan (blue) in the central regions of the PAR2A IVDs relative to MCCM injury and uninjured controls (Figure 5A). These results suggest that PAR2A does not have any detrimental effects on viability but decreases the expression of angiogenic factors while promoting matrix accumulation.

### Human *in vitro* Monolayer Therapeutic Model

**FIGURE 6:**
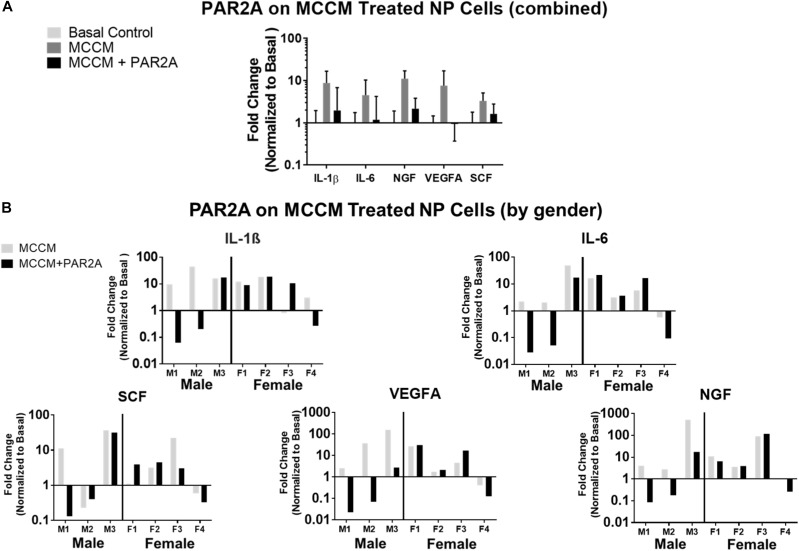
**(A)** Histogram of average fold change in qRT-PCR gene expression in human NP cells (*N* = 7) treated with MCCM and MCCM+PAR2A normalized to basal untreated controls. **(B)** Histograms of fold changes in qRT-PCR gene expression in human NP cells for individual donors normalized to basal control where left (M1, M2, M3) are males and right (F1, F2, F3, F4) are females.

To further assess the therapeutic potential and clinical translation of PAR2A we evaluated the effects of PAR2A on human cells *in vitro*. PAR2A consistently down-regulated chemokine (SCF), inflammatory (IL-1β; IL-6), neurotrophic (NGF) and angiogenic (VEGFA) markers in human NP IVD cells treated with MCCM compared to human NP cells treated with MCCM alone although this was not statistically significant (Figure 6A). When presenting the data as individual human samples, biological differences were observed when assessing gender, where at least 2/3 male NP cells treated with MCCM appeared to respond to PAR2A with down-regulation of the majority of inflammatory and neurovascular factors assessed. When assessing female NP samples, only 1/4 demonstrated a notable response to treatment with PAR2A across a majority of the genes assessed (Figure 6B), suggesting gender differences may influence the therapeutic potential of the biologics targeted in the degenerate IVD, however, more validation is needed before any specific conclusions can be drawn.

## Discussion

Immune cells, more specifically MCs, have been well documented as a key contributor to the pathology of many musculoskeletal disorders related to joint degradation such as rheumatoid arthritis (Woolley, 2003). Additionally, these cells are known to play a role in several chronic pain conditions such as migraines, inflammatory arthritis and irritable bowel syndrome (O’Sullivan et al., 2000; Nigrovic and Lee, 2005; Levy et al., 2007). Our previously published data has shown significant evidence to support a role for MCs in the pathogenesis of painful IVD degeneration, with increased numbers of MCs in diseased IVD tissue from patients with DBP relative to healthy controls (Wiet et al., 2017). Furthermore, we demonstrated that IVD cells express MC chemoattractants such as SCF and MCs interact with healthy bovine IVD cells to up-regulate catabolic and inflammatory markers such ADAMTS5 and IL-6. Interestingly, soluble factors from bovine IVD cells were able to induce MC degranulation and up-regulation of VEGFA in MCs (Wiet et al., 2017). However, a number of questions still remain with respect to the role of MCs in human DBP and the potential to therapeutically target these pathological effects. Specifically, do IVD cells promote migration of MCs, how do MC mediators such as tryptase interact with human IVD cells and tissue, at the gene and matrix level, and what are the potential pathways down-stream of these effects and can they be therapeutically modulated. The answers to these questions form the basis of the work described here.

To understand the role of MCs in DBP, it is important to investigate how these cells may infiltrate the IVD microenvironment, an environment that is typically immune-privileged. As such, we investigated the ability of soluble factors from human IVD cells to recruit HMC-1 MCs using an *in vitro* migration assay system. Derivatives of the hematopoietic precursor lineage, MCs are unique in their ligand-based maturation at a tissue target site rather than in the stem cell microenvironment, and as such chemoattractant ligands, such as SCF, are crucial in their recruitment. The HMC-1 cell line in particular has been shown to adequately express the C-kit receptor for SCF as well as migrate in a dose dependent manner to SCF. Importantly, they were shown to migrate non-randomly toward SCF rather than random chemokinesis, making it an adequate model to study this mechanism of migration (Nilsson et al., 1994a,b). Our study demonstrates that when MCs are exposed to IVDCM, migration of MCs is significantly upregulated compared to BMC (negative control) groups. Furthermore, this effect was not seen when comparing AC cell control media, ACCM, suggesting this effect is likely specific. We have previously demonstrated that SCF is expressed by human IVD cells from all regions of the IVD in health and disease and is a likely candidate for mediating these effects, however, further work is needed to validate this effect and the role SCF may play (Wiet et al., 2017). Indeed, SCF is a major chemoattractant for MCs and their progenitors *in vivo* (Okayama and Kawakami, 2006) and to our knowledge our study is the first to demonstrate that soluble factors from IVD cells can induce MC migration *in vitro*.

Our previous studies have demonstrated that MCs release soluble factors that can modulate IVD cell behavior and phenotype and one candidate mediator is tryptase, yet the specific effect of tryptase and potential down-stream pathways have not been elucidated. Our study demonstrates that healthy human NP cells, when treated with rhTryptase, exhibit significant upregulation of SCF, and this effect appears to be dose dependent. This suggests that tryptase may help facilitate IVD-induced MC migration into the IVD creating a positive feed-back loop and promoting a chronic inflammatory response whereby more MCs migrate into the diseased IVD and degranulate, releasing their catabolic and inflammatory mediators further enhancing this pathogenic cycle of disease. This mechanistic model is supported by our previous finding of more pronounced SCF expression in the NP region at the protein level (IHC) (Wiet et al., 2017). In this study we also observed significant up-regulation of pro-angiogenic factor VEGFA in NP cells in response to treatment with rhTryptase. This finding is complementary to our previous studies showing significantly increased VEGFA secretion by MCs when exposed to IVDCM from degenerate NP cells relative to basal control, pointing toward MC/IVD interactions mediating pro-angiogenic effects in the painful IVD (Wiet et al., 2017). While the possibility of native MC contamination of IVD cells cannot be definitively excluded given the presence of MCs in cadaveric disc tissue as cited in our previous work (Wiet et al., 2017) MCs are non-adherent cells therefore monolayer culture, used for initial IVD expansion, would very likely exclude MCs from culture. The painful degenerate IVD is characterized by neo-vascular invasion (Freemont et al., 2002; Lee et al., 2011; Stefanakis et al., 2012; Binch et al., 2015; Lama et al., 2018) and given how MCs are associated with angiogenic processes in multiple tissues and pathologies (Kneilling et al., 2007; De Palma et al., 2017; Longo et al., 2018) suggests that tryptase may be associated and enhance this process in the diseased IVD.

In assessing the full spectrum of potential effects that tryptase may have on the IVD there are many considerations beyond the chemokines SCF and VEGFA alone. Tryptase has been shown to activate the zymogen form of MMP3 and MMP13, and these activated proteases can directly degrade aggrecan in femoral cartilage explants (Magarinos et al., 2013). Aggrecan represents the principle structural proteoglycan of the NP, suggesting that tryptase may have similar effects with respect to the ECM of the IVD. Our current study shows that when treated with rhTryptase, isolated NP explants from healthy human IVD specimens released significantly more sulfated glycosaminoglycan (GAG) into the medium, suggesting that MCs can induce degradation of the ECM in the NP via tryptase. This effect was not observed for the AF. These results suggest that, as in the studies by Magarinos et al. (2013), tryptase can enhance degradation of proteoglycan which is relevant to degradation of the IVD. Healthy NP tissue is aggrecan rich, and previous studies have demonstrated that aggrecan is inhibitory to both nerve ingrowth and endothelial cell migration and adhesion (Johnson et al., 2002, 2005). Degradation of proteoglycans, such as aggrecan, by MC enzymes, such as tryptase, could facilitate neurovascular ingrowth into the painful IVD. Specifically, it has been well documented that neo-vascularization and the ingrowth of nociceptive neurons is a marker of DBP progression, and this effect is related to sites of matrix degradation (Smith et al., 2011; Stefanakis et al., 2012; Lama et al., 2018).

To determine potential therapeutic strategies in the context of tryptase, it was crucial to identify the downstream mechanism by which it can affect the IVD cells themselves. Causes of cellular changes related to tryptase however, have not been well characterized. Tryptase has been well documented as a potent activator of PAR2 (Molino et al., 1997) and PAR2 activation has been cited in many catabolic, inflammatory, and pain inducing pathways. Notable among these was the finding that PAR2 activation is sufficient to induce matrix degradation in osteoarthritic cartilage, significantly stimulating catabolic and inflammatory factors such as MMP13 and IL-1β, respectively. Further, this process was also seen to be self-promoting following onset, with findings that PAR2 is upregulated in diseased tissue and by IL-1β (Boileau et al., 2007). In order to validate the potential of tryptase/PAR2 interactions in IVD pathology, it was important to confirm and quantify the expression of PAR2 in all regions of the human IVD. Our findings validated the work of Iida et al. (2009) that demonstrated clear and levels of expression of PAR2 for non-diseased IVD cells in the NP and AF regions. However, to our knowledge we also show for the first time PAR2 expression in the CEP region with levels that are comparable to that of the NP. Our IHC data, confirmed by western blot, in healthy samples supports a potential role for tryptase/PAR2 interactions in the IVD pathology.

Functional PAR2 activation involves proteolytic cleavage of the amino terminus and uncovering of a tethered ligand interacting with exterior facial loops of the membrane bound protein, leading to a crystalline activation state (Nystedt et al., 1994). The present study sought to investigate this interaction as a therapeutic target using a PAR2 antagonist (PAR2A), the small molecule oligopeptide inhibitor FSLLRY-NH_2._ This antagonist functions by blocking this terminal cleavage and has been cited to abate PAR2 pathological effects in several studies such as Chen et al. (2015) and their treatment of neuropathic pain. The skeletally mature bovine IVD is an excellent tool and model for investigating degeneration of the IVD *ex vivo* and for therapeutic screening of targets related to DBP (Purmessur et al., 2013b; Illien-Jünger et al., 2014; Grant et al., 2016) and as such to elucidate the tryptase/PAR2 interaction in disease we developed an *ex vivo* bovine organ culture model representing injury and exposure to MC related soluble factors. Our findings demonstrated that PAR2A had no effect on native cell viability and also demonstrated enhanced matrix regeneration following injury as shown by increased histological blue staining of aggrecan and accumulation of tissue following culture of injured samples in MCCM, when compared to injured controls. Furthermore, in AF tissue the gene expression of VEGFA was significantly downregulated when comparing the same groups. As previously discussed, VEGFA is crucial to the neo-vascularization process and has been shown to promote neurovascular ingrowth in degraded AF tissue (Stefanakis et al., 2012). As such, down-regulation of VEGFA and increased matrix synthesis with treatment of PAR2A highlights its therapeutic potential for restoring structure/function to the IVD while reducing neo-angiogenic processes. However, this “proof of concept” study requires further validation with a larger cohort of IVDs and for a longer time-frame to determine whether effects are sustained.

To further investigate the therapeutic potential of PAR2A on IVD cells we conducted a preliminary *in vitro* human study utilizing PAR2A to screen IVD cells considered most physiologically and clinically relevant to DBP. It is important to note that in order to better elaborate on the microenvironment of a MC modulated disease state in the native IVD, MCCM in its entirety was utilized. This MCCM includes a variety of mediators derived from the granules such as catabolic enzyme ADAMTS5 and inflammatory/pain-associated factors IL-1β, TNFα, VEGFA, NGF and Substance P among others (De Schepper et al., 2010), and tryptase content has been previously validated in these MCCM samples (Wiet et al., 2017) as well as in the present study design. Our study demonstrated that MCCM induced a consistent inflammatory and pathological response in human NP cells. Treatment with PAR2A seemed to abate these effects in a fairly consistent manner, however, when all donors were combined, these results were not statistically significant. Use of human IVD samples for drug screening is highly clinically relevant, yet due to the innate nature of human variability, effects are often dismissed as there exists a pool of non-responders versus responders (Madian et al., 2012). Indeed, DBP in clinical practice has been shown to be enigmatic in its presentation with underlying risk factors not always evident and fully elaborated on in the field. One study in particular found that even with MRI imaging cross-sectional analysis of asymptomatic persons aged 60 or older, 36% had disc herniations, 21% had spinal stenosis and more than 90% had degenerated and or bulging discs (Boden et al., 1990) demonstrating that in many ways our cross-referential understanding of the manifestation of DBP is still emerging. As such, we sought to understand how sample demographics could potentially influence the response to PAR2A treatment. Interestingly, when we examined the effects of PAR2A as a function of male vs. female derived samples we found that in the majority of the assessed genetic markers, at least 2/3 male derived samples responded to PAR2A with down-regulation of the MCCM induced effects. This trend was not nearly as pronounced for female samples, with only 1/4 of these samples demonstrating a consistent response to PAR2A in the assessed genes. It has been shown that gender differences are observed for DBP clinically such as Bartynski et al. (2013) finding that in men, injury related DBP represented 83% of all injury related cases examined with DBP while 65% of progressive onset associated DBP cases being female patients. Additionally, it was found that in cases of progressive onset, significantly more severely degenerative discs in females were reported (65%) when compared to male patients (27%) even though across the entire sample, cases of severely degenerative discs were nearly identical to full-thickness radial fissures (47.3% vs. 52.7%), a type of IVD internal derangement and typical clinical indicator of back pain. It was the conclusion of this group that gender differences may be relevant to the consideration of back pain origin, treatment, and response to treatment. These findings seem to be corroborated by more recent studies such as that Mosley et al. (2019) examining gender differences in a rat AF puncture degenerative model. The study found that in this degenerative model, female rats responded to puncture with decreased collagen organization and density, fibril diameter, and increased molecular damage when compared to males. Furthermore, it was suggested that differences in females are likely to contribute to difficulty in healing, and more pronounced pain in female clinical DBP cases. The application of these findings among others in the context of the present study’s findings opens the possibility that demographic considerations are of potential importance in analysis of therapeutic options. While these considerations are potentially promising in the progress toward developing non-surgical intervention therapies for DBP, it is important to note that significantly more human samples are needed to fully validate and elaborate on these differing outcomes across demographics seen within the present study before any definitive conclusions can be drawn. As such, we report only the potential of PAR2A as a treatment option, and one that should be assessed with consideration of gender among other demographic considerations. Further investigation of these factors is needed to expand upon our preliminary investigation and hopefully elucidate any practical applications there may be to the benefit of the very diverse affected DBP patient population. As such, further work by our group includes longitudinal investigation, expansion of sample size, assessment of age, gender, race and species effects among other factors, to verify the validity of PAR2A as a potential therapeutic strategy for the treatment of DBP.

## Significance

This study highlights chemotactic effects of IVD cells on MCs, evidence toward mast cell/PAR2 interactions regulating inflammatory and angiogenic markers of discogenic back pain, and PAR2A as a potential therapeutic strategy specifically targeting mast cell/PAR2 interactions in IVD pathophysiology.

## Ethics Statement

IRB: 2018H0424 for patient articular cartilage tissue. Autopsy samples from the Cooperative Human Tissue Network are IRB exempted.

## Author Contributions

All authors contributed to the conception and design of the study, or acquisition of data, or analysis and interpretation of data, drafted the article or revised it critically for important intellectual content, and approved the final version to be submitted. DP took responsibility for the integrity of the work as a whole, from inception to finished article.

## Conflict of Interest Statement

The authors declare that the research was conducted in the absence of any commercial or financial relationships that could be construed as a potential conflict of interest.
