# Supplementary figures and images for: Mast Cell/Proteinase Activated Receptor 2 (PAR2) Mediated Interactions in the Pathogenesis of Discogenic Back Pain
Source: Front Cell Neurosci. 2019 Jul 5;13:294. doi: 10.3389/fncel.2019.00294 (PMC6625229; doi:10.3389/fncel.2019.00294)

Supplemental Fig. 1:

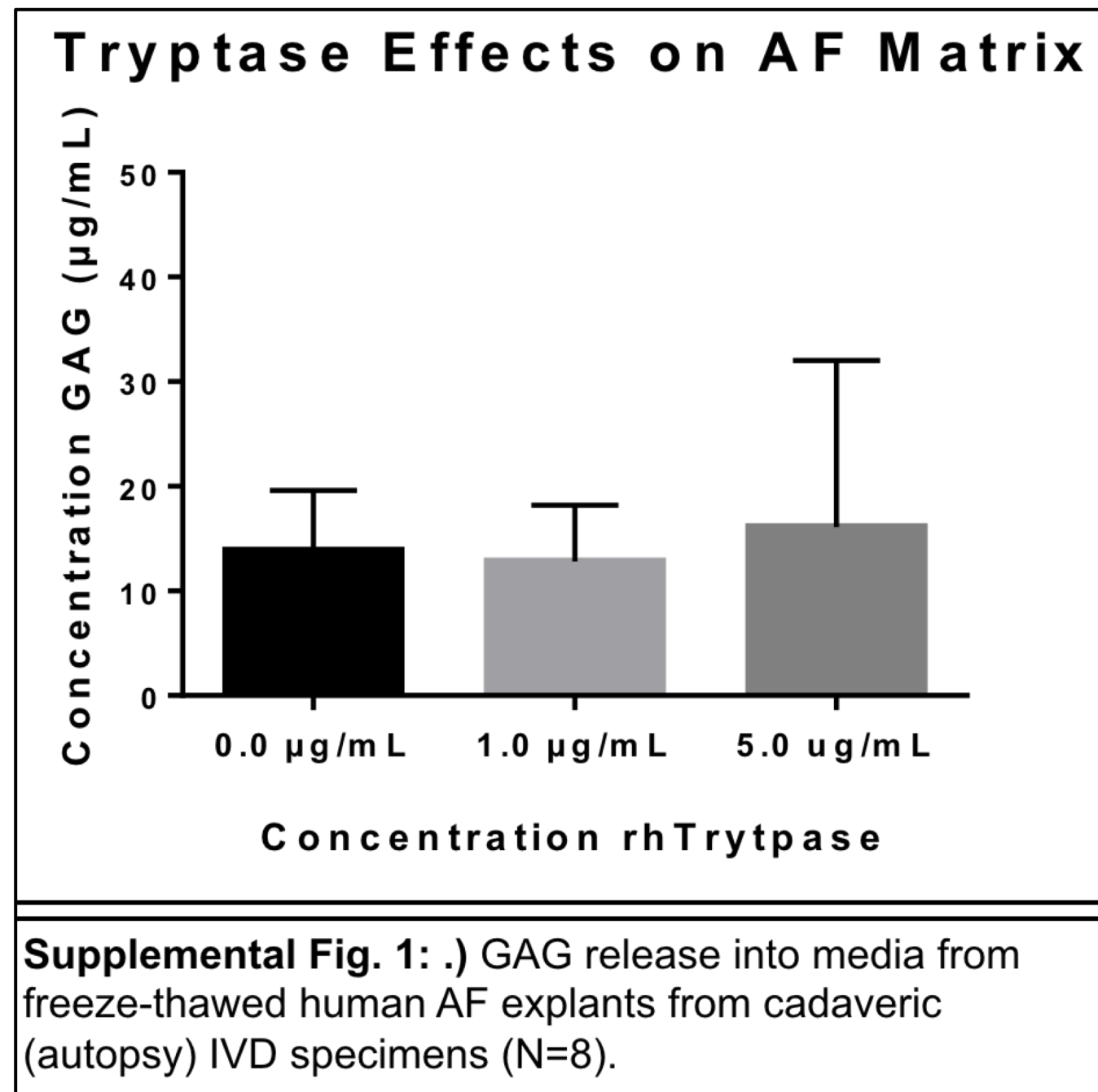

Supplement: Supplementary file 1 [file Data_Sheet_1.PDF]
