# Supplementary material for: Mast Cell/Proteinase Activated Receptor 2 (PAR2) Mediated Interactions in the Pathogenesis of Discogenic Back Pain
Source: Front Cell Neurosci. 2019 Jul 5;13:294. doi: 10.3389/fncel.2019.00294 (PMC6625229; doi:10.3389/fncel.2019.00294)
Supplement: Supplementary file 2 [file Data_Sheet_2.PDF]

Supplemental Fig. 2:

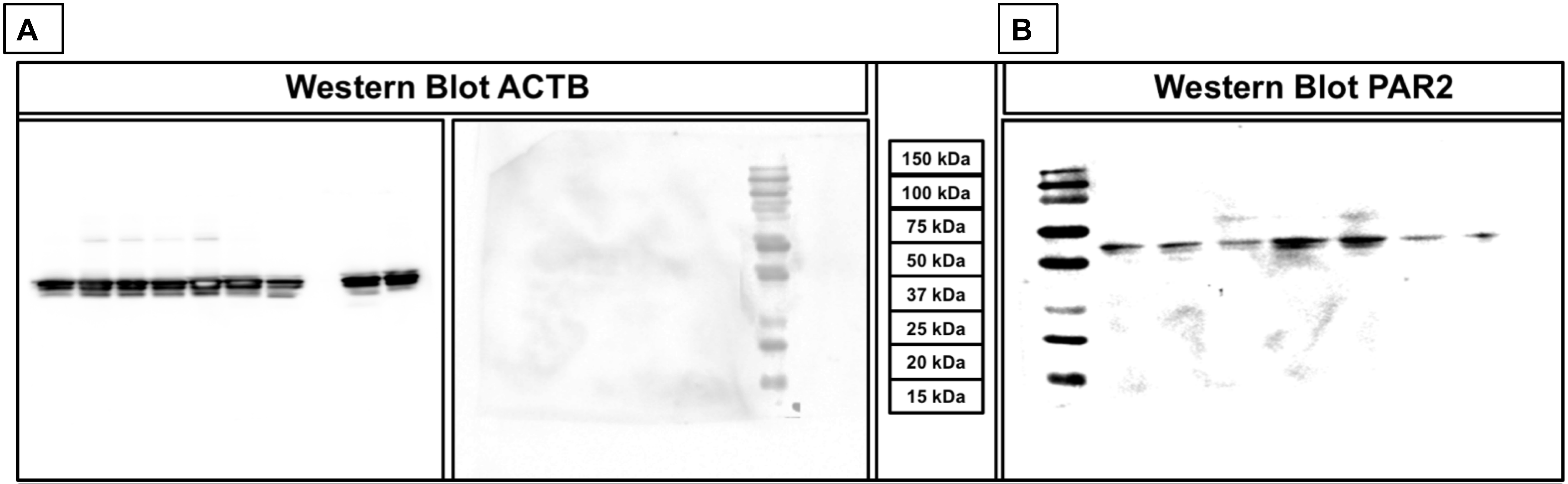

Supplementary Figure 2: Western blot (original gels and ladders with labeling. **A.)** CTB: Ladder image was not imposed onto original image due to Azure Imager error at time of imaging. PAR2 and ACTB ran on same electrophoresis run in separate gels and transferred to different membranes due to relativity in size. Image correlates with figure 4 (B). Both Western Images have been modified the way (horizontal elongation to align with Figure 4.) **B.)** Loading Map of Ladder (L) in lane 1 and respective samples with unedited images western Blot using Azure Imager. Blot used for Figure 4 (B). Both Western Images have been modified the way (horizontal elongation to align with Figure 4.)
