# Supplementary material for: Mast Cell/Proteinase Activated Receptor 2 (PAR2) Mediated Interactions in the Pathogenesis of Discogenic Back Pain
Source: Front Cell Neurosci. 2019 Jul 5;13:294. doi: 10.3389/fncel.2019.00294 (PMC6625229; doi:10.3389/fncel.2019.00294)
Supplement: Supplementary file 3 [file Data_Sheet_3.PDF]

Supplemental Table 1: Relevant Gene Descriptions

Supplementary Table 1: Gene Descriptions	
**GENE**	**Description**
PAR2 (F2RL1)	GPCR activated by amino terminus cleavage by trypsin like proteases. Show to be important in blood flow, inflammatory response and innate/adaptive immune systems.
SCF (KITLG)	Ligand (KIT Ligand) of the tyrosine-kinase receptor. Pleiotropic factor involved in hematopoietic precursor stem cell development and mast cell migration/development specifically.
VEGFA	Growth factor promoting vascular endothelial cell migration and proliferation. Essential for normal and pathological angiogenesis.
NGF	Growth factor regulating growth and proper differentiation of sensory neurons.
IL-1B	Proprotein (proteolytic activation) cytokine associated with immune response. Important mediator proliferation, apoptosis, and both normal inflammatory responses and inflammatory pain hypersensitivity.
IL-6	Cytokine involved in inflammatory response in acute and chronic conditions. Ability of endogenous pyrogen activity in autoimmune responses.
MMP3	Proteolytically activated proproteins involved in breakdown of extracellular matrix (various collagens and proteoglycans) during tissue remodeling responses in normal and pathological physiology.
MMP13	Member of same matrix metalloproteinase family as MMP3 and as relevant to study functions as described above for MMP3.
